# Editorial: Decoding cell fate: the critical roles of extracellular vesicles

**DOI:** 10.3389/fcell.2025.1615951

**Published:** 2025-05-08

**Authors:** Yongqiang Chen, Saeid Ghavami, Paul C. Park, Joy Irobi

**Affiliations:** ^1^ Manitoba Chemosensory Biology Research Group, Department of Oral Biology, Dr. Gerald Niznick College of Dentistry, University of Manitoba, Winnipeg, MB, Canada; ^2^ Children’s Hospital Research Institute of Manitoba, University of Manitoba, Winnipeg, MB, Canada; ^3^ Department of Human Anatomy and Cell Sciences, Max Rady College of Medicine, University of Manitoba, Winnipeg, MB, Canada; ^4^ Paul Albrechtsen Research Institute, CancerCare Manitoba, University of Manitoba, Winnipeg, MB, Canada; ^5^ Akademia Śląska, Katowice, Poland; ^6^ Department of Pathology, Max Rady College of Medicine, University of Manitoba, Winnipeg, MB, Canada; ^7^ Department of Immunology and Infections, Biomedical Research Institute, Hasselt University, Hasselt, Belgium

**Keywords:** extracellular vesicles, exosome, cell survival, cell death, autophagy, apoptosis, microRNA, osteoarthritis

## 1 Background

Once considered passive byproducts of cellular metabolism, extracellular vesicles (EVs) have emerged as finely tuned intercellular communication messengers that orchestrate biological processes, such as immune responses and disease progression (Kumar et al., 2024; Yanez-Mo et al., 2015; Yim et al., 2020). These nano- and micro-sized vesicles range from 30 to 5,000 nm and mainly include exosomes, microvesicles, and apoptotic bodies; they are secreted by prokaryotic and eukaryotic cells, transporting a wide range of biologically active molecules, such as proteins, lipids, and nucleic acids (Mobarak et al., 2024).

While traditional studies have focused primarily on intracellular pathways and direct environmental stressors as key regulators of cell fate, this Research Topic shifts the lens toward the emerging influence of EVs in dictating whether a cell survives or dies and how disease can be treated. Recent advances suggest that EVs are not merely passive information conveyors but active participants in the dynamic balance between cellular resilience and vulnerability (Xia et al., 2022; Sulek, 2024). They mediate protective functions under stress, modulate autophagy, apoptosis, and immune responses, and have been implicated in a range of pathophysiological conditions, such as neurodegeneration, cancer, fibrosis, and autoimmune disease (Wilczak et al., 2024; Zhang et al., 2025).

## 2 New findings in this Research Topic

This Topic has collected one Brief Research Report article and three review articles. Gade et al. reported paradigm-shifting original findings related to secretory mitophagy. Under oxidative stress, when damaged mitochondria exceed the degradation capacity of the lysosome, cancer cells adopt a surprising survival mechanism. Rather than undergoing degradation via the lysosome, dysfunctional mitochondria are expelled out of the cell through EVs enriched with the mitophagy regulator PINK1 (PTEN (phosphatase and tensin homolog deleted on chromosome 10)-induced kinase 1). The EV-mediated removal of “toxic” defective mitochondria can help prevent cancer cells from dying. The study identified a novel, EV-mediated resilience pathway in tumor cells that could be exploited to sensitize cancer cells to oxidative therapies.

Longfei et al. comprehensively reviewed the therapeutic implications of exosomes in osteoarthritis (OA), a degenerative joint disease manifested by joint dysfunction and pain, which are caused by cartilage breakdown, bone spur formation, and synovial inflammation. The study revealed a dual role of EVs in relieving or exacerbating OA, depending on their sources. Mesenchymal stem cell (MSC)-derived exosomes could benefit OA treatment by promoting chondrocyte proliferation, alleviating inflammation, and inhibiting cell death (apoptosis). The types of microRNAs (miRNAs) in exosomes from synovial fibroblasts determine the impact of exosomes on cartilage repair for OA treatment: miRNAs like miR-19b-3p and miR-106b can exacerbate articular cartilage damage, whereas other miRNAs such as miR-182-5p, miR-214-3p, miR 126-3P, and miR-142-5p can promote articular cartilage repair. Similarly, healthy chondrocyte exosomes could help cartilage repair; in contrast, degenerative chondrocyte exosomes may play an opposite role. Exosomes from anti-inflammatory M2 macrophages or pro-inflammatory M1 macrophages might benefit or worsen OA treatment, respectively. Interestingly, EVs from Traditional Chinese Medicine (TCM) herbs (e.g., *Morinda Officinalis* and *Rhizoma Drynariae*) can potentially promote OA treatment.

The roles of MSC-derived EVs (MSC-EVs) in disease treatment are also reviewed by Su et al. and Wang et al.. Su et al. revealed that MSC-EVs could help treat many diseases, such as diabetes and neurodegenerative diseases, via activating the antioxidant and anti-inflammatory nuclear factor erythroid 2-related factor 2 (Nrf2)/heme oxygenase-1 (HO-1) axis pathway. Wang et al. summarized the roles of MSC-EVs in the treatment of systemic sclerosis (SSc), a complex disease characterized by vasculopathy, immune dysfunction, and fibrosis. MSC-EVs could promote vascular repair, reduce immunogenicity and fibrosis, and cross the blood-brain barrier, facilitating SSc treatment.

## 3 Perspectives

EVs have become hot research topics in disease diagnosis and treatment. Articles collected in this Research Topic highlight the multifaceted roles of EVs in cellular decision-making. From cancer cell survival, antioxidant defense, and immune modulation to OA and SSc treatment, EVs are more than vesicular bystanders—they are functional effectors capable of tipping the scales between degeneration and regeneration, as well as disease progression and therapeutic success.

**FIGURE 1:**
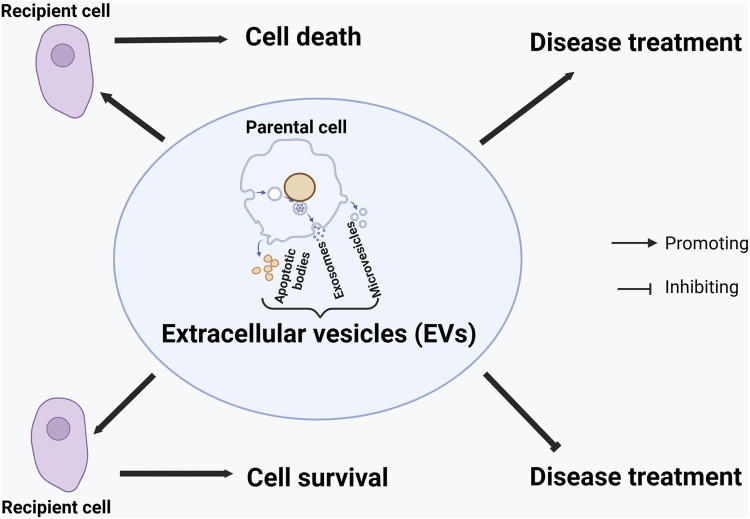
Graphical model of the effects of extracellular vesicles on cell survival or death and disease treatment. The effects are context-dependent on the types of (parental and recipient) cells, the types of extracellular vesicles (EVs), the composition of EVs, and the disease.

EVs from parental cells can promote cell survival or death in recipient cells (Sanwlani and Gangoda, 2021) (Figure 1). This Research Topic reveals a new mechanism that parental cells can use their EVs to transport “toxic” intracellular components (e.g., damaged mitochondria) outside to prolong survival. More studies are needed to evaluate whether different “toxic” elements can be expelled out of the cell to support survival.

EVs can also play a dual role (promoting or worsening) in disease treatment, depending upon the context (Figure 1). They could have opposite effects on treating the same disease (e.g., OA) if derived from different sources of cells in the same organism. Regarding the same disease (e.g., OA), EVs from healthy cells (e.g., healthy chondrocytes) could help its treatment, whereas those from “diseased” cells (e.g., degenerative chondrocytes) do not. This observation might be extrapolated to other diseases, although more studies are needed to verify the hypothesis. When EVs are derived from the same type of cells (e.g., synovial fibroblasts), their components (e.g., the types of miRNAs) could contribute to different outcomes (e.g., promoting cartilage repair or damage). The possibility of using TCM-derived EVs for OA treatment suggests that EVs from different kingdoms or species of organisms can be explored to treat human diseases.

As the field progresses, understanding the precise bioactive cargo, delivery mechanisms, and context-specific effects of EVs will be essential. The promise they hold across disciplines, from oncology to rheumatology to regenerative medicine, invites a rethinking of how we approach complex diseases. The Topic provides an overview of the current breakthroughs and lays a compelling foundation for future research that positions EVs at the center of next-generation diagnostics and therapies.
